# Supplementary material for: Developmental disruption and restoration of brain synaptome architecture in the murine Pax6 neurodevelopmental disease model
Source: Nat Commun. 2022 Nov 11;13:6836. doi: 10.1038/s41467-022-34131-w (PMC9652404; doi:10.1038/s41467-022-34131-w)
Supplement: Supplementary file 1 — Supplementary Information [file 41467_2022_34131_MOESM1_ESM.pdf]

Supplementary Information

Developmental disruption and restoration of brain synaptome architecture in the murine Pax6 neurodevelopmental disease model

Laura Tomas-Roca, Zhen Qiu, Erik Fransén, Ragini Gokhale, Edita Bulovaite, David J. Price, Noboru H. Komiyama, Seth G.N. Grant.

Contents

Supplementary Figures (1-4)

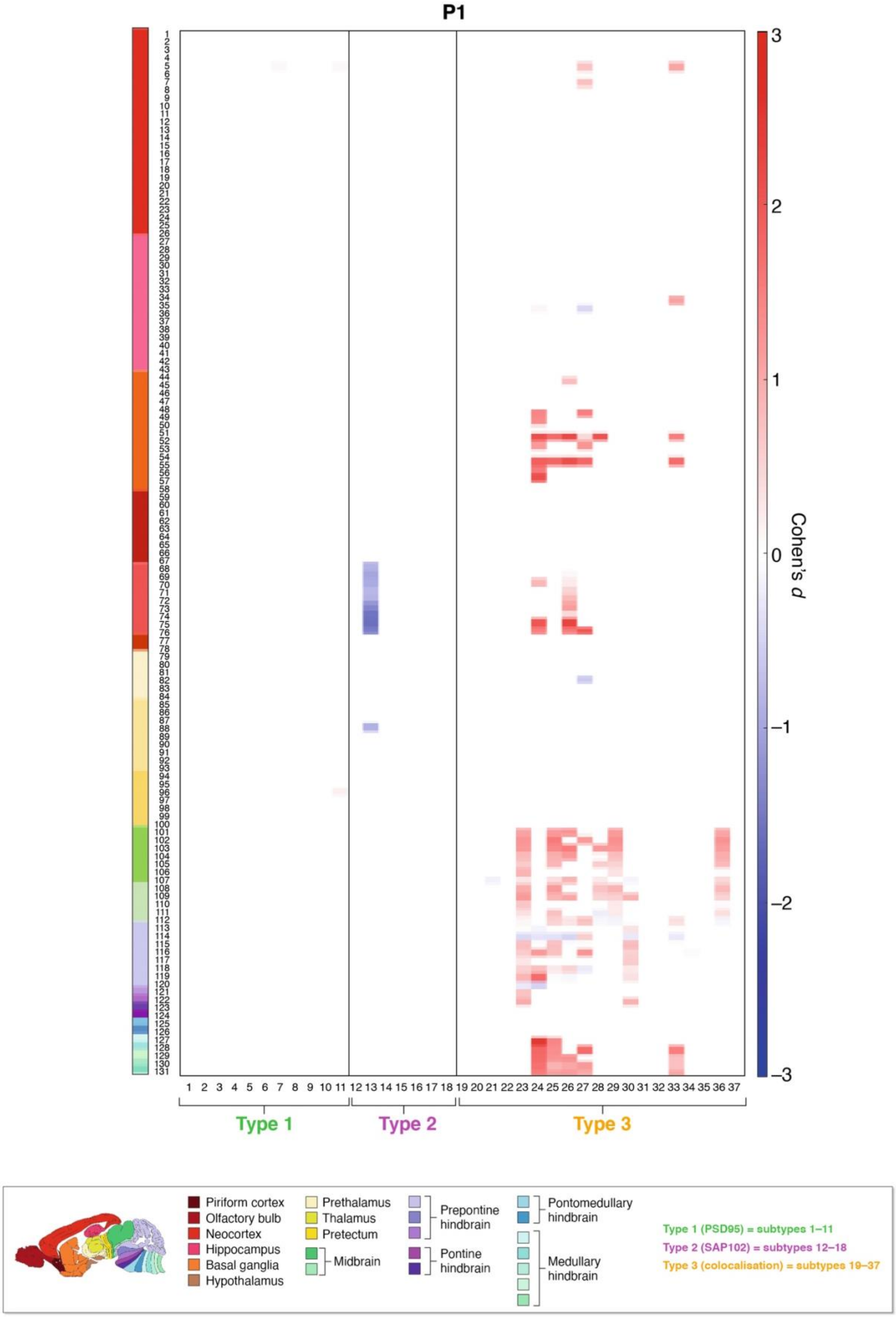

P1
Cohen's d
Type 1
Type 2
Type 3
Piriform cortex
Olfactory bulb
Neocortex
Hippocampus
Basal ganglia
Hypothalamus
Prethalamus
Thalamus
Pretectum
Midbrain
Prepontine hindbrain
Pontine hindbrain
Pontomedullary hindbrain
Medullary hindbrain
Type 1 (PSD95) = subtypes 1–11
Type 2 (SAP102) = subtypes 12–18
Type 3 (colocalisation) = subtypes 19–37

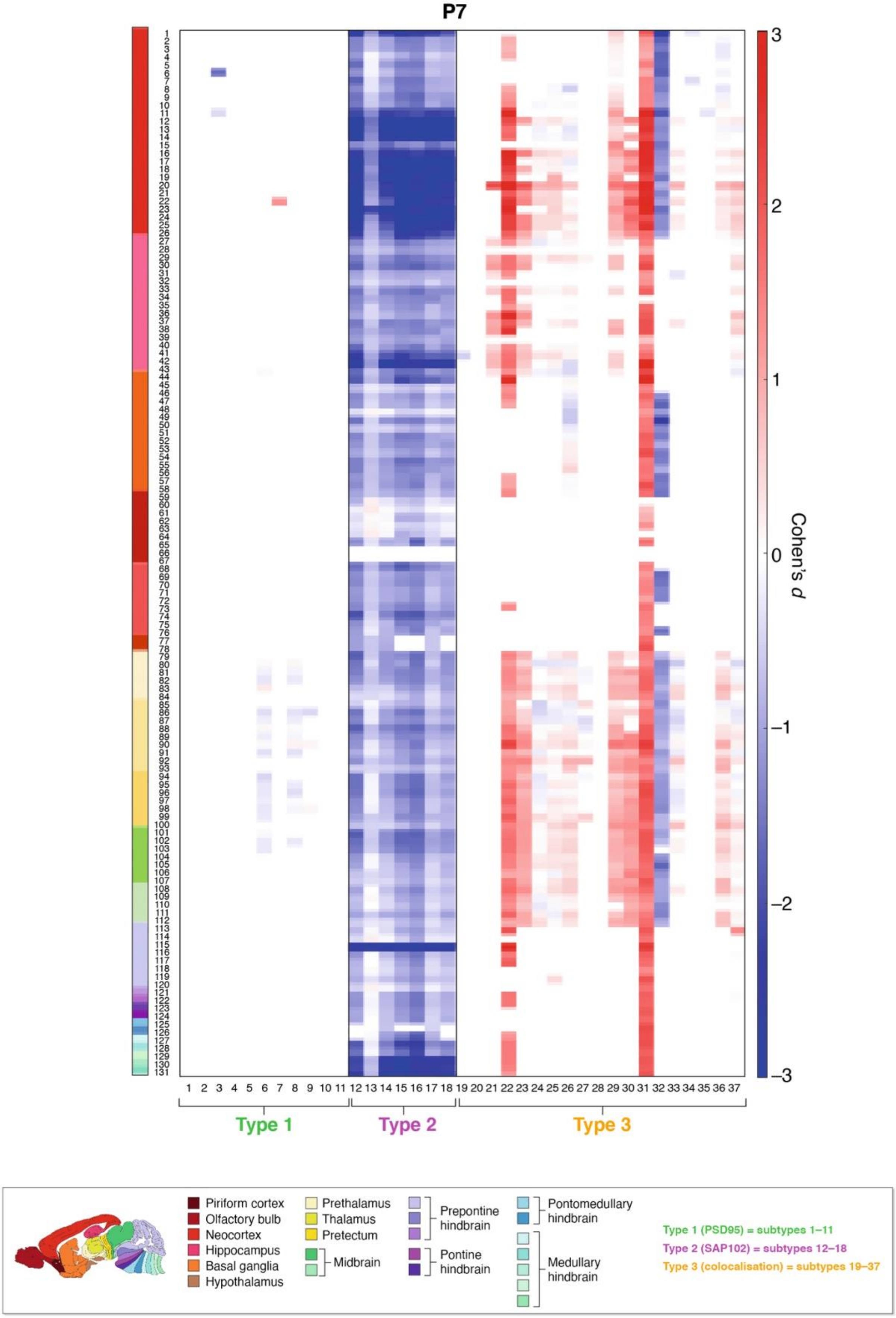
P7
Cohen's d
3
2
1
0
−1
−2
−3
Type 1
Type 2
Type 3
Piriform cortex
Olfactory bulb
Neocortex
Hippocampus
Basal ganglia
Hypothalamus
Prethalamus
Thalamus
Pretectum
Midbrain
Prepontine hindbrain
Pontine hindbrain
Pontomedullary hindbrain
Medullary hindbrain
Type 1 (PSD95) = subtypes 1–11
Type 2 (SAP102) = subtypes 12–18
Type 3 (colocalisation) = subtypes 19–37

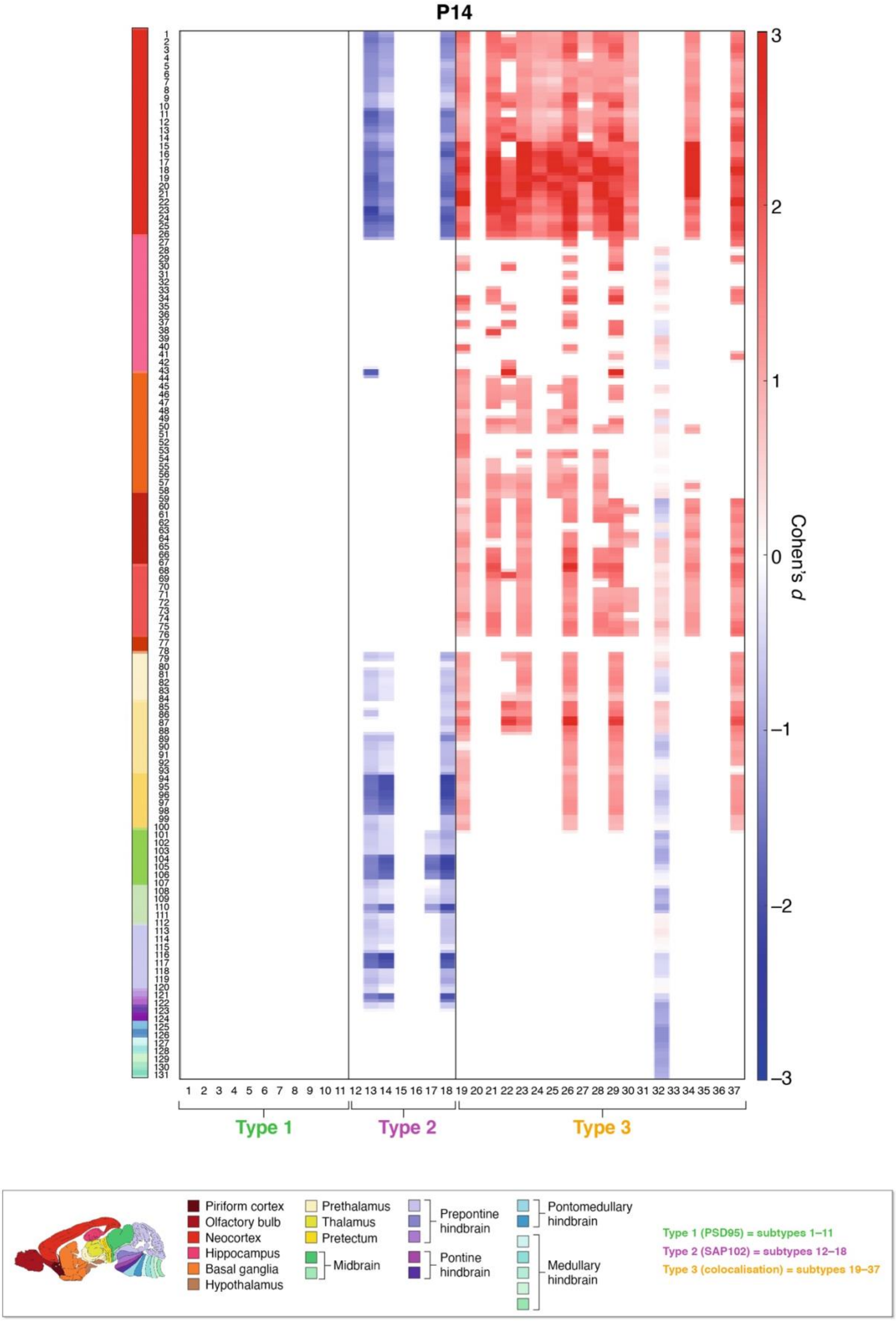
P14
Cohen's d
3
2
1
0
−1
−2
−3
Type 1
Type 2
Type 3
Piriform cortex
Olfactory bulb
Neocortex
Hippocampus
Basal ganglia
Hypothalamus
Prethalamus
Thalamus
Pretectum
Midbrain
Prepontine hindbrain
Pontine hindbrain
Pontomedullary hindbrain
Medullary hindbrain
Type 1 (PSD95) = subtypes 1–11
Type 2 (SAP102) = subtypes 12–18
Type 3 (colocalisation) = subtypes 19–37

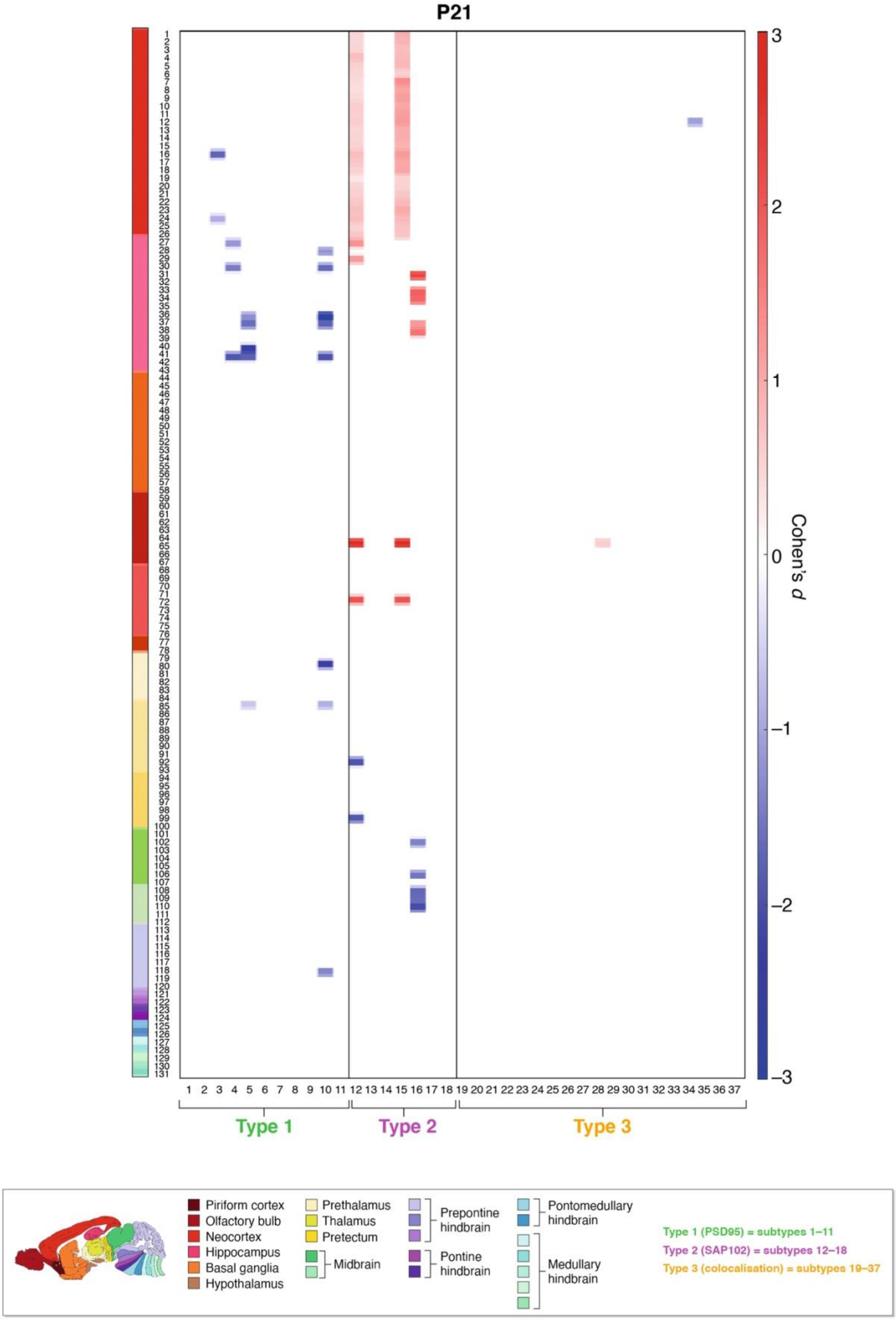
P21
Cohen's d
Type 1
Type 2
Type 3
Piriform cortex
Olfactory bulb
Neocortex
Hippocampus
Basal ganglia
Hypothalamus
Prethalamus
Thalamus
Pretectum
Midbrain
Prepontine hindbrain
Pontine hindbrain
Pontomedullary hindbrain
Medullary hindbrain
Type 1 (PSD95) = subtypes 1–11
Type 2 (SAP102) = subtypes 12–18
Type 3 (colocalisation) = subtypes 19–37

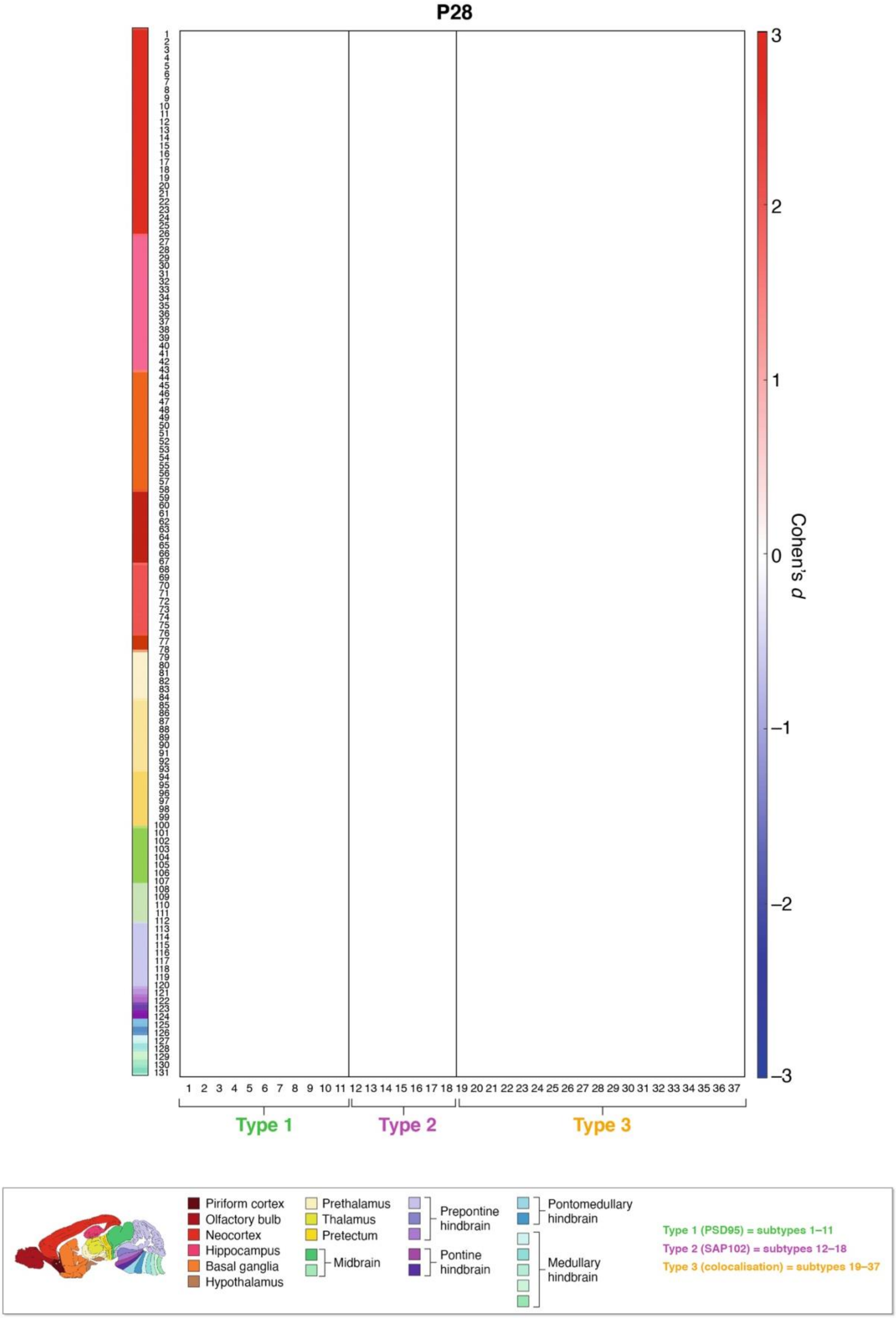

P28
Cohen's d
3
2
1
0
−1
−2
−3
Type 1
Type 2
Type 3
Piriform cortex
Olfactory bulb
Neocortex
Hippocampus
Basal ganglia
Hypothalamus
Prethalamus
Thalamus
Pretectum
Midbrain
Prepontine hindbrain
Pontine hindbrain
Pontomedullary hindbrain
Medullary hindbrain
Type 1 (PSD95) = subtypes 1–11
Type 2 (SAP102) = subtypes 12–18
Type 3 (colocalisation) = subtypes 19–37

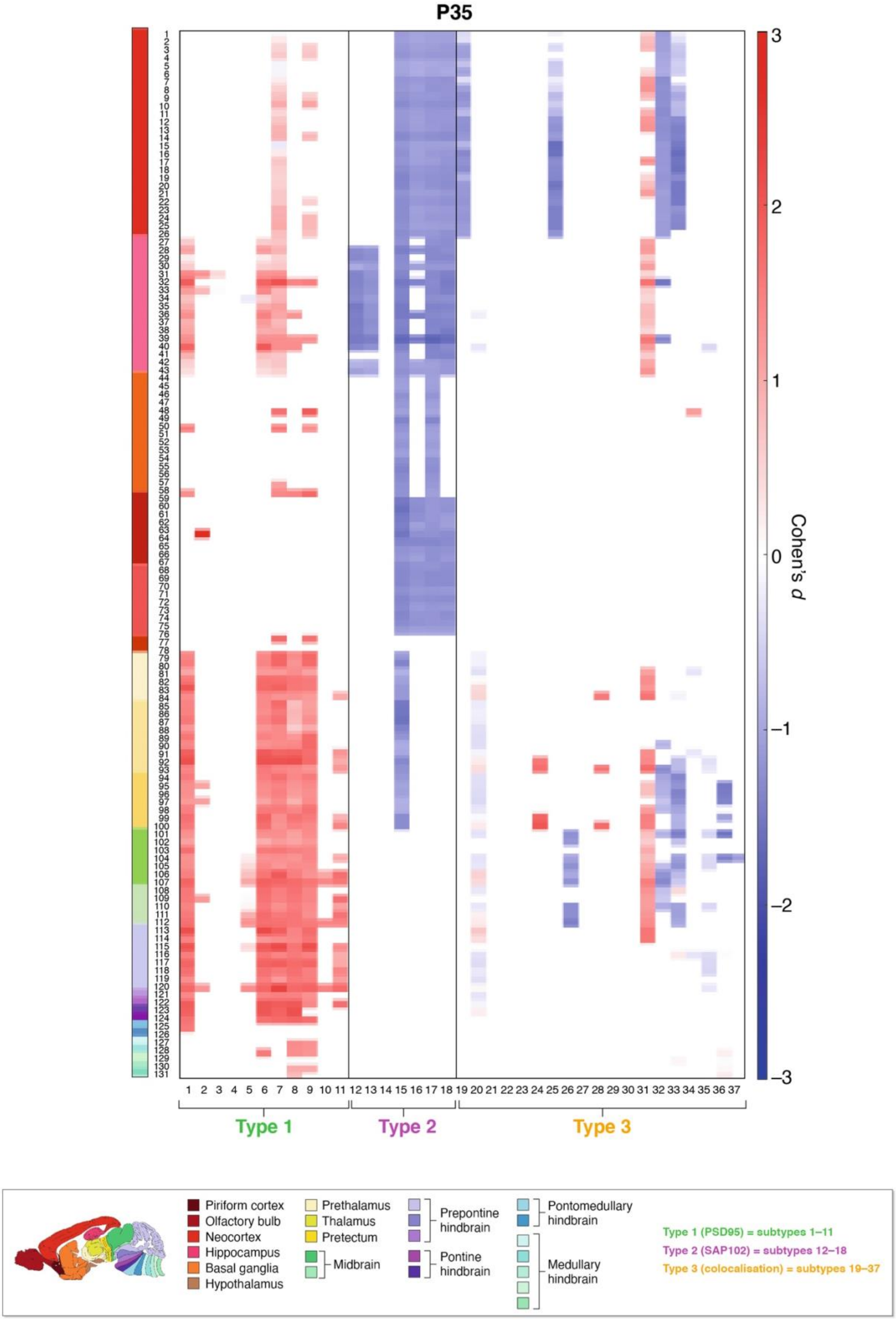
P35
Cohen's d
Type 1
Type 2
Type 3
Piriform cortex
Olfactory bulb
Neocortex
Hippocampus
Basal ganglia
Hypothalamus
Prethalamus
Thalamus
Pretectum
Midbrain
Prepontine hindbrain
Pontine hindbrain
Pontomedullary hindbrain
Medullary hindbrain
Type 1 (PSD95) = subtypes 1–11
Type 2 (SAP102) = subtypes 12–18
Type 3 (colocalisation) = subtypes 19–37

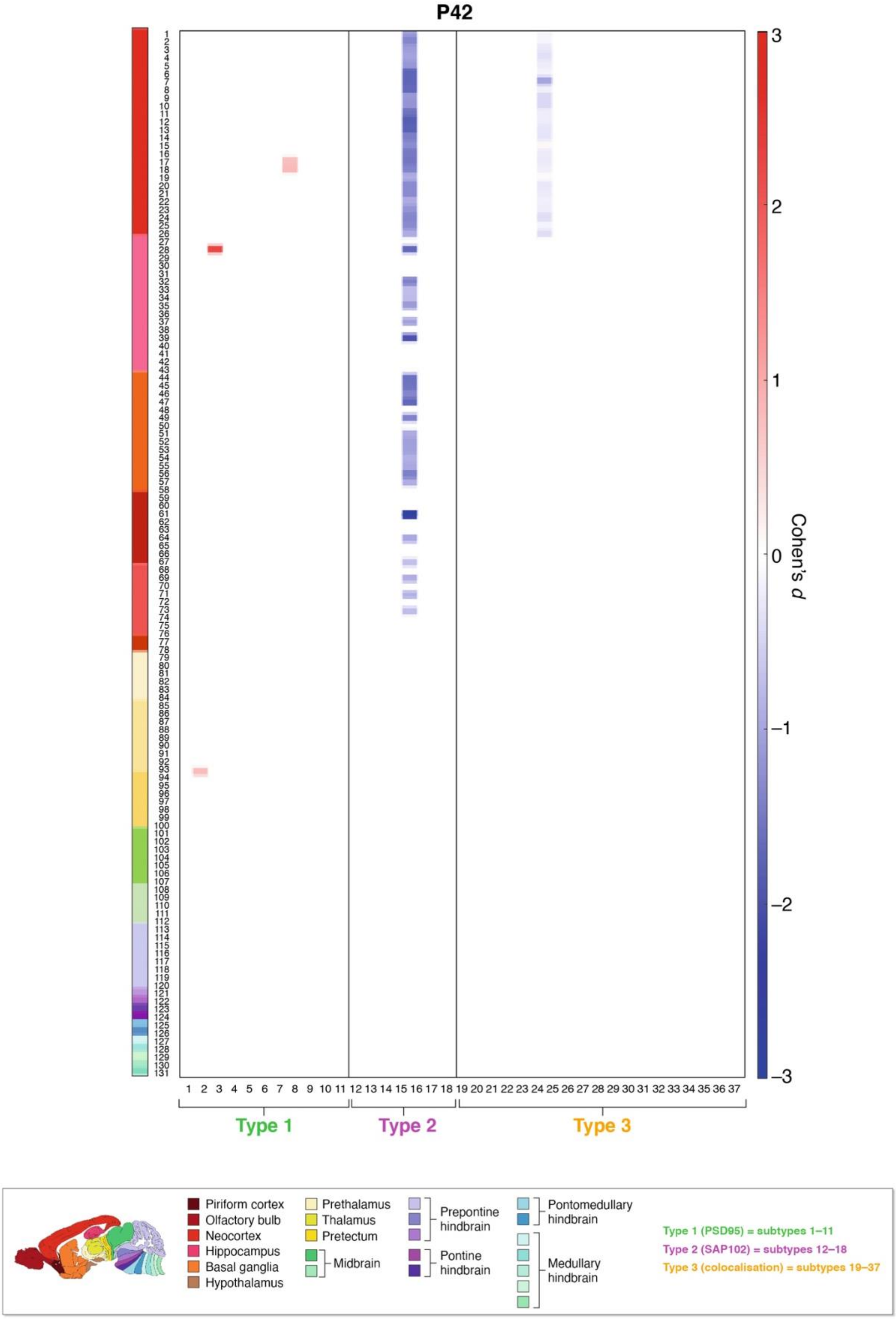
P42
Cohen's d
3
2
1
0
−1
−2
−3
Type 1
Type 2
Type 3
Piriform cortex
Olfactory bulb
Neocortex
Hippocampus
Basal ganglia
Hypothalamus
Prethalamus
Thalamus
Pretectum
Midbrain
Prepontine hindbrain
Pontine hindbrain
Pontomedullary hindbrain
Medullary hindbrain
Type 1 (PSD95) = subtypes 1–11
Type 2 (SAP102) = subtypes 12–18
Type 3 (colocalisation) = subtypes 19–37

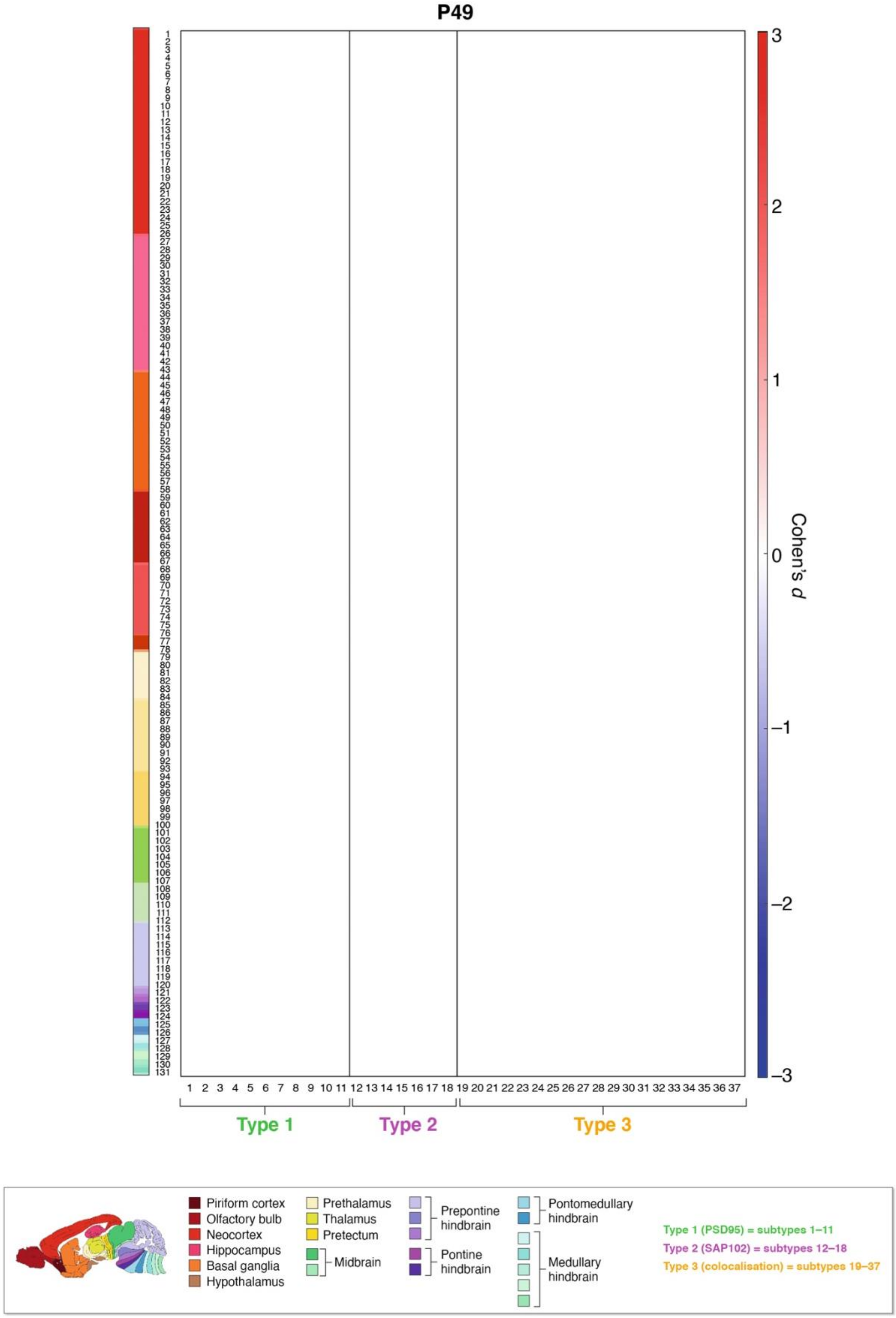
P49
Cohen's d
3
2
1
0
−1
−2
−3
Type 1
Type 2
Type 3
Piriform cortex
Olfactory bulb
Neocortex
Hippocampus
Basal ganglia
Hypothalamus
Prethalamus
Thalamus
Pretectum
Midbrain
Prepontine hindbrain
Pontine hindbrain
Pontomedullary hindbrain
Medullary hindbrain
Type 1 (PSD95) = subtypes 1–11
Type 2 (SAP102) = subtypes 12–18
Type 3 (colocalisation) = subtypes 19–37

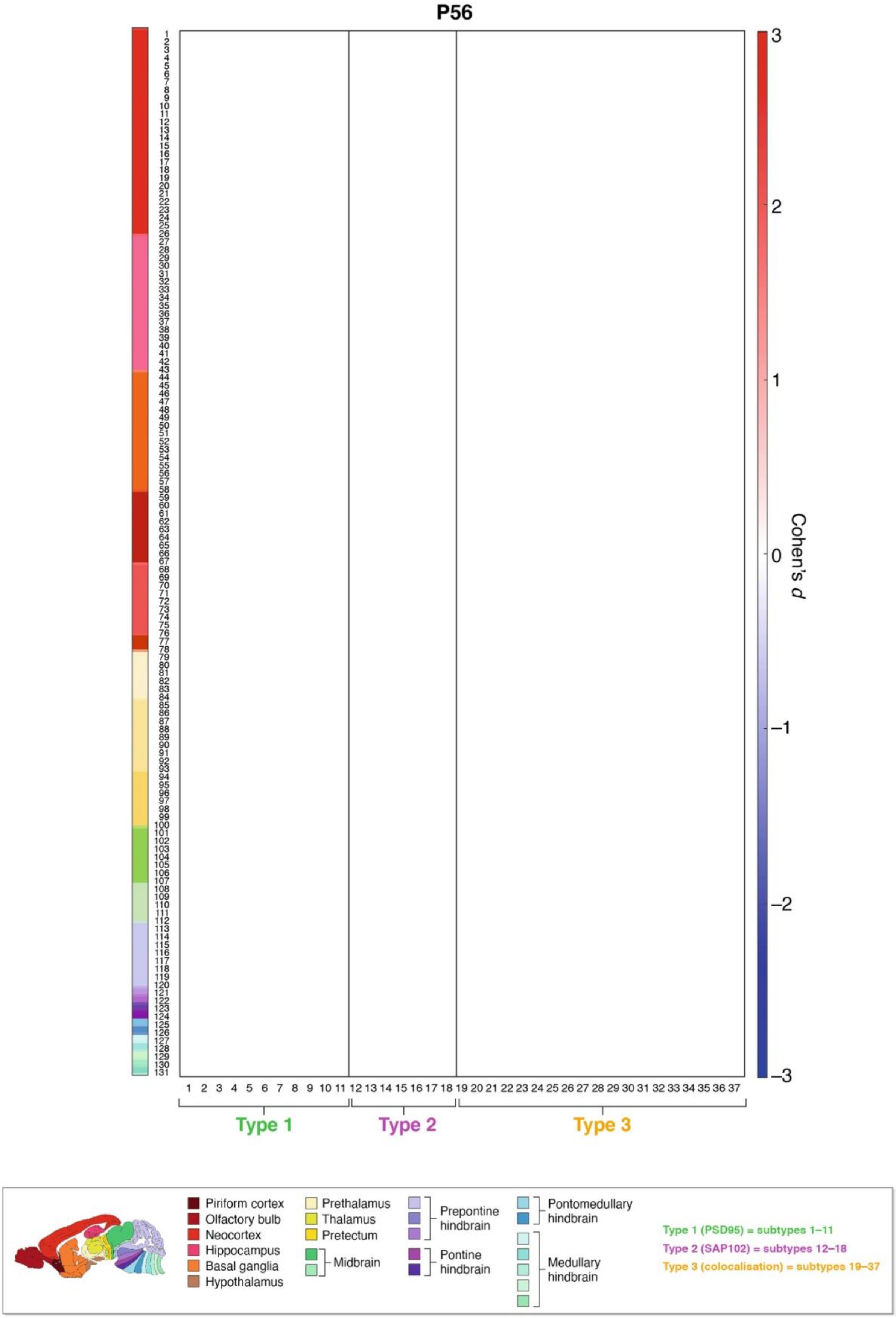

Figure S1. Brain synaptome phenotypes transiently emerge and repair during postnatal development of *Pax6*$^{+/-}$ mice.

The difference (Cohen's *d*) in synapse type and subtype density in 131 brain subregions at nine ages between birth and maturity between *Pax6*$^{+/-}$ and control mice. Significantly different (P<0.05, Bayesian test, Benjamini-Hochberg corrected) subregions are shown.

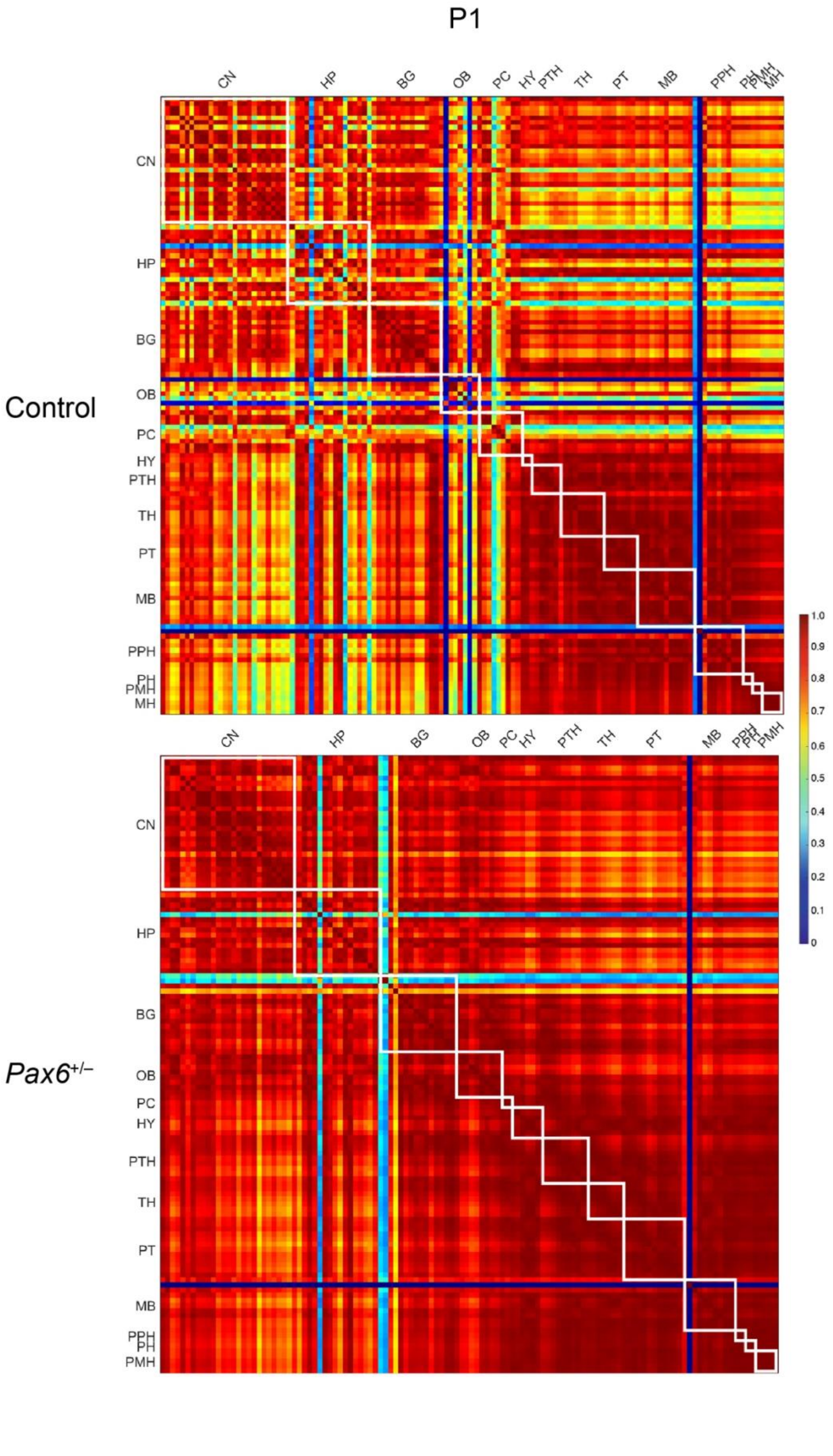
P1
Control
Pax6+/–
CN
HP
BG
OB
PC
HY
PTH
TH
PT
MB
PPH
PH
PMH
MH
1.0
0.9
0.8
0.7
0.6
0.5
0.4
0.3
0.2
0.1
0

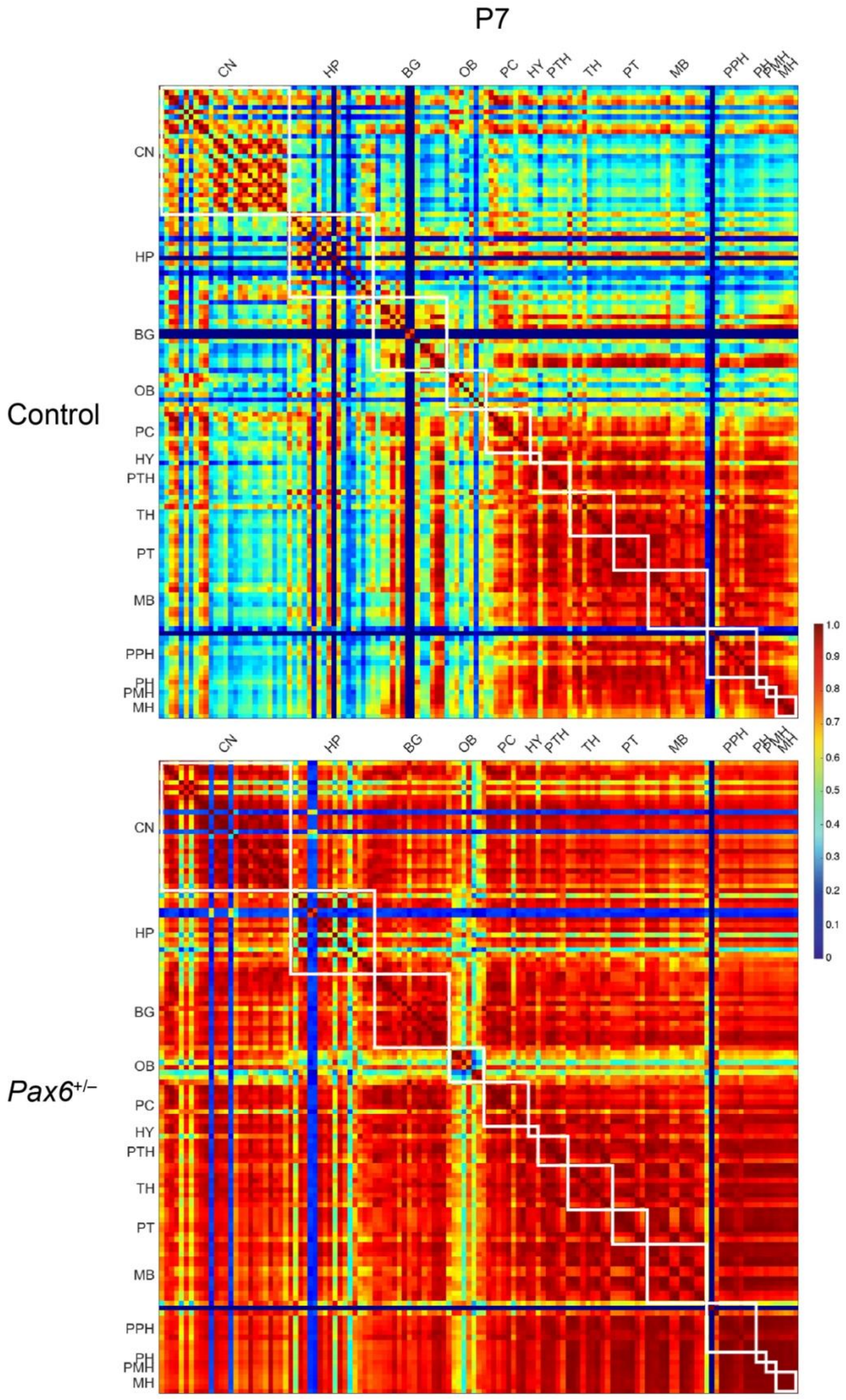

P7
Control
Pax6+/–
CN
HP
BG
OB
PC
HY
PTH
TH
PT
MB
PPH
PH
PMH
MH
1.0
0.9
0.8
0.7
0.6
0.5
0.4
0.3
0.2
0.1
0

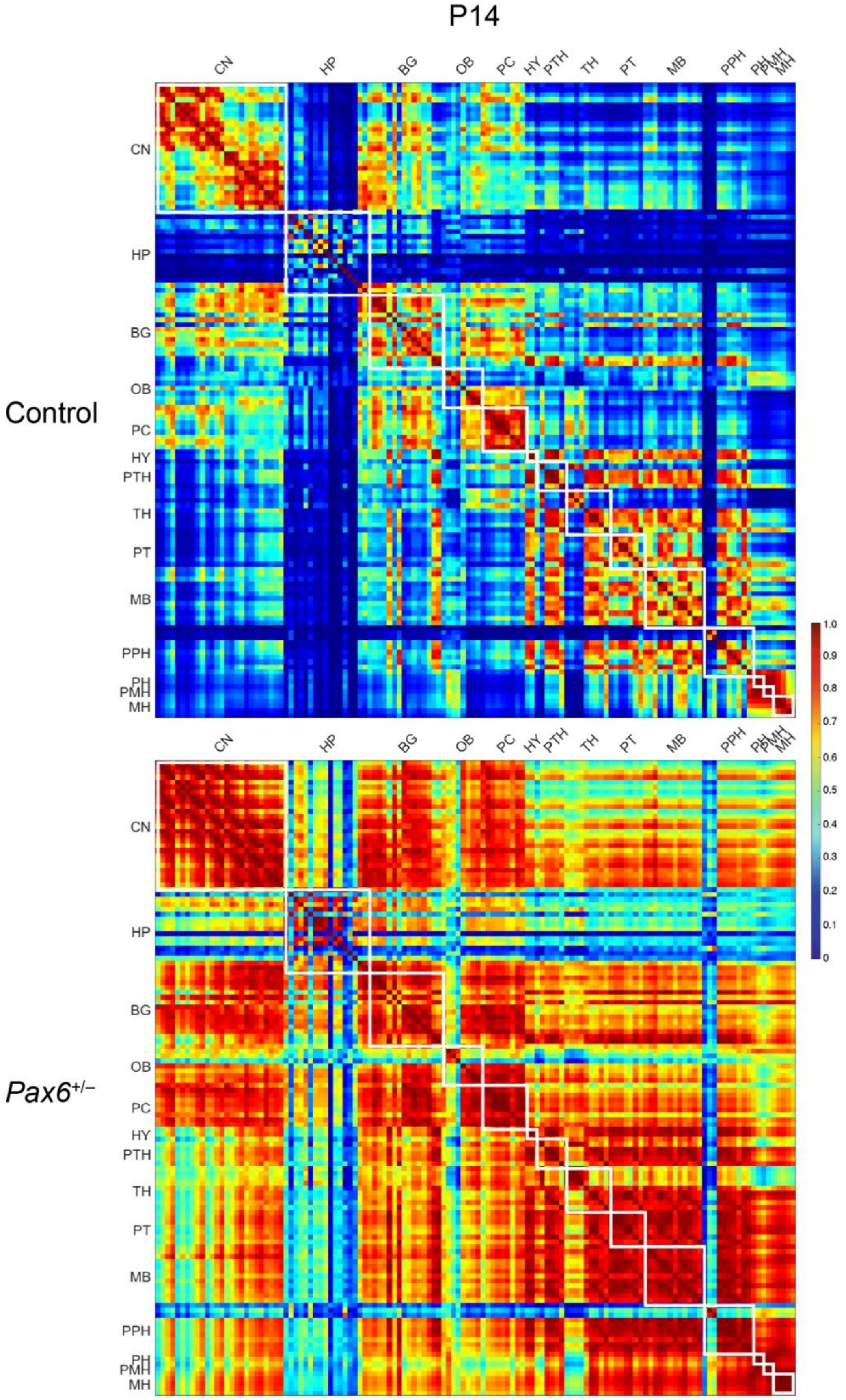
P14
Control
Pax6+/−
CN
HP
BG
OB
PC
HY
PTH
TH
PT
MB
PPH
PH
PMH
MH
1.0
0.9
0.8
0.7
0.6
0.5
0.4
0.3
0.2
0.1
0

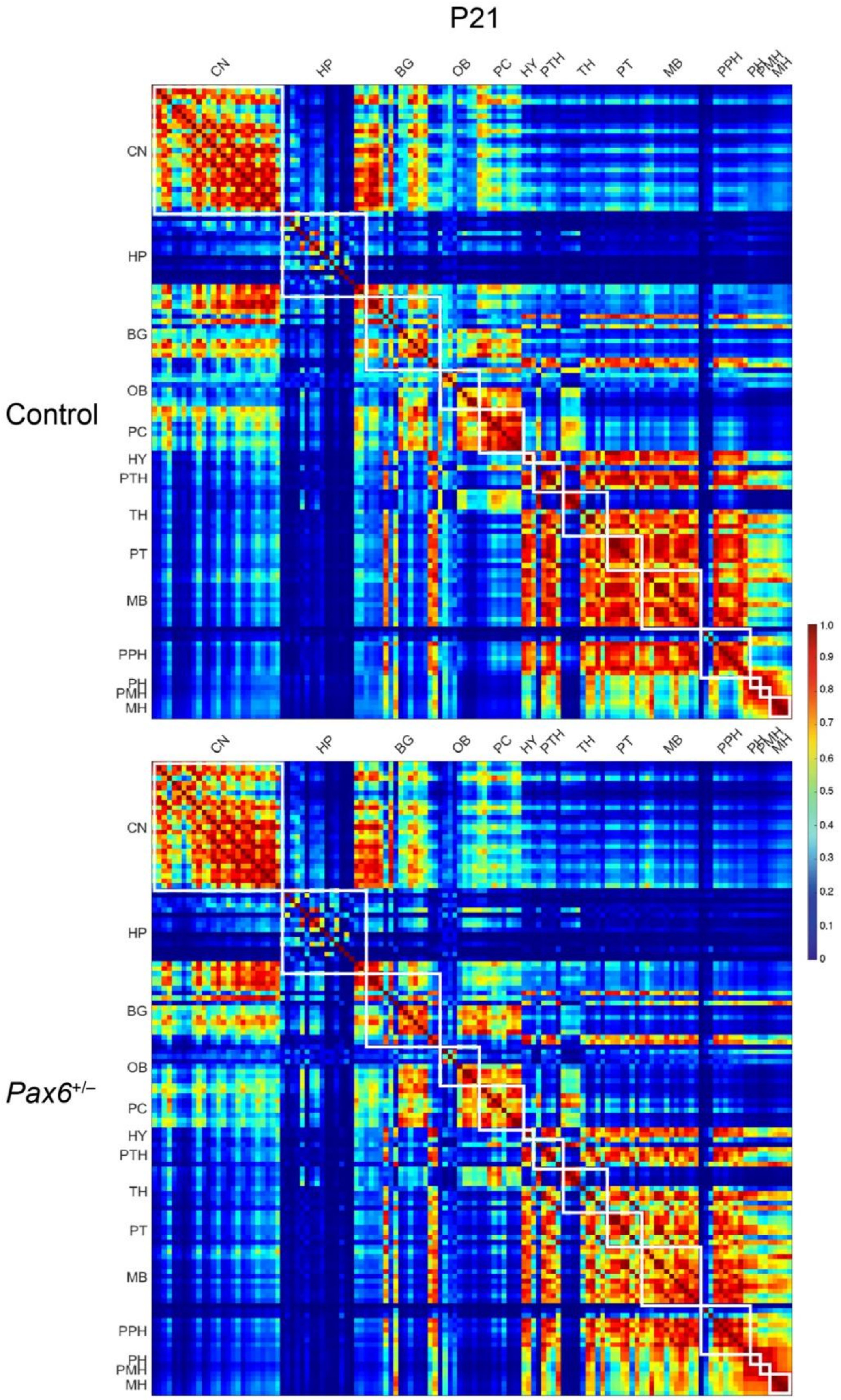
P21
Control
Pax6+/–
CN
HP
BG
OB
PC
HY
PTH
TH
PT
MB
PPH
PH
PMH
MH
1.0
0.9
0.8
0.7
0.6
0.5
0.4
0.3
0.2
0.1
0

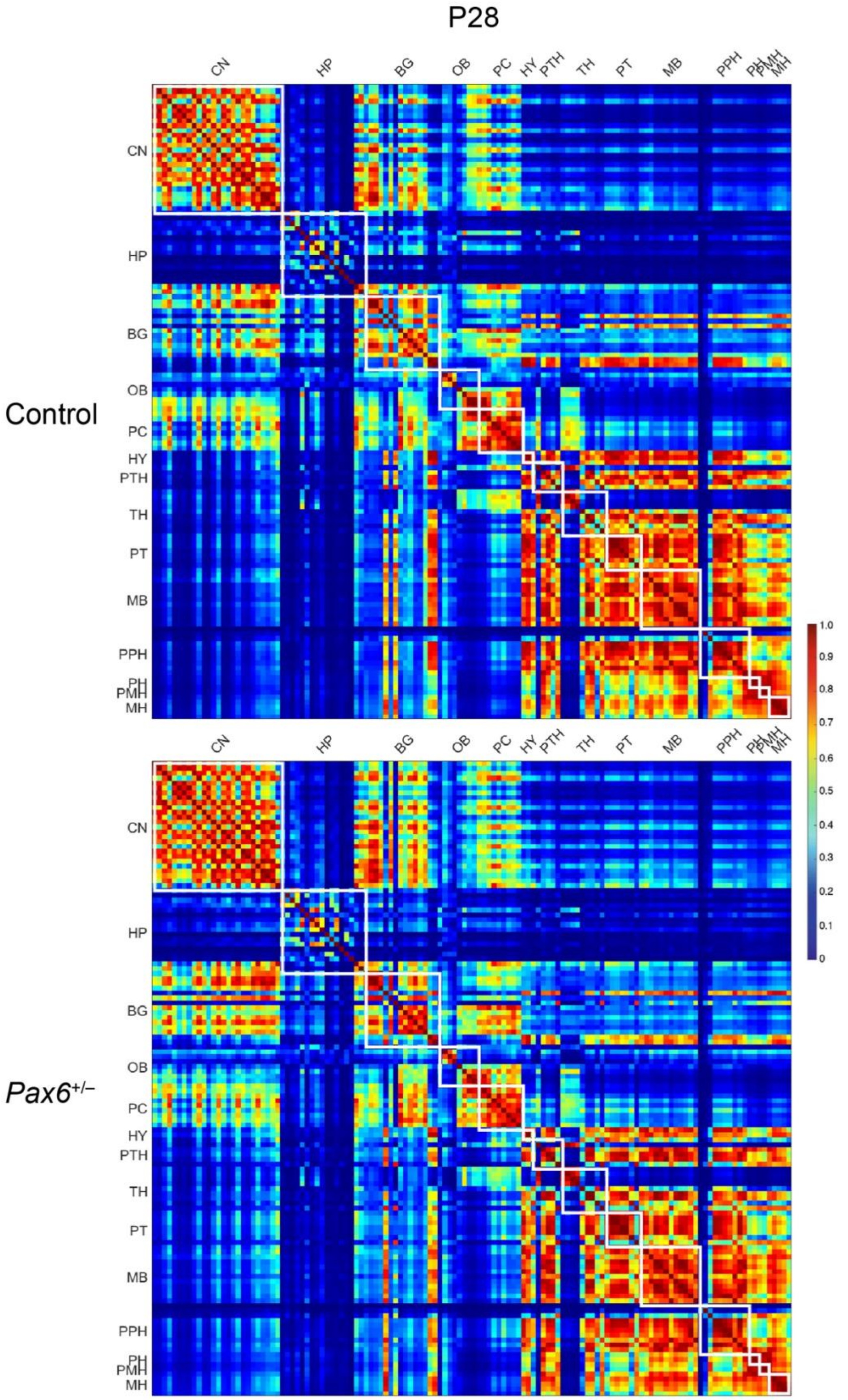
P28
Control
Pax6+/−
CN
HP
BG
OB
PC
HY
PTH
TH
PT
MB
PPH
PH
PMH
MH
1.0
0.9
0.8
0.7
0.6
0.5
0.4
0.3
0.2
0.1
0

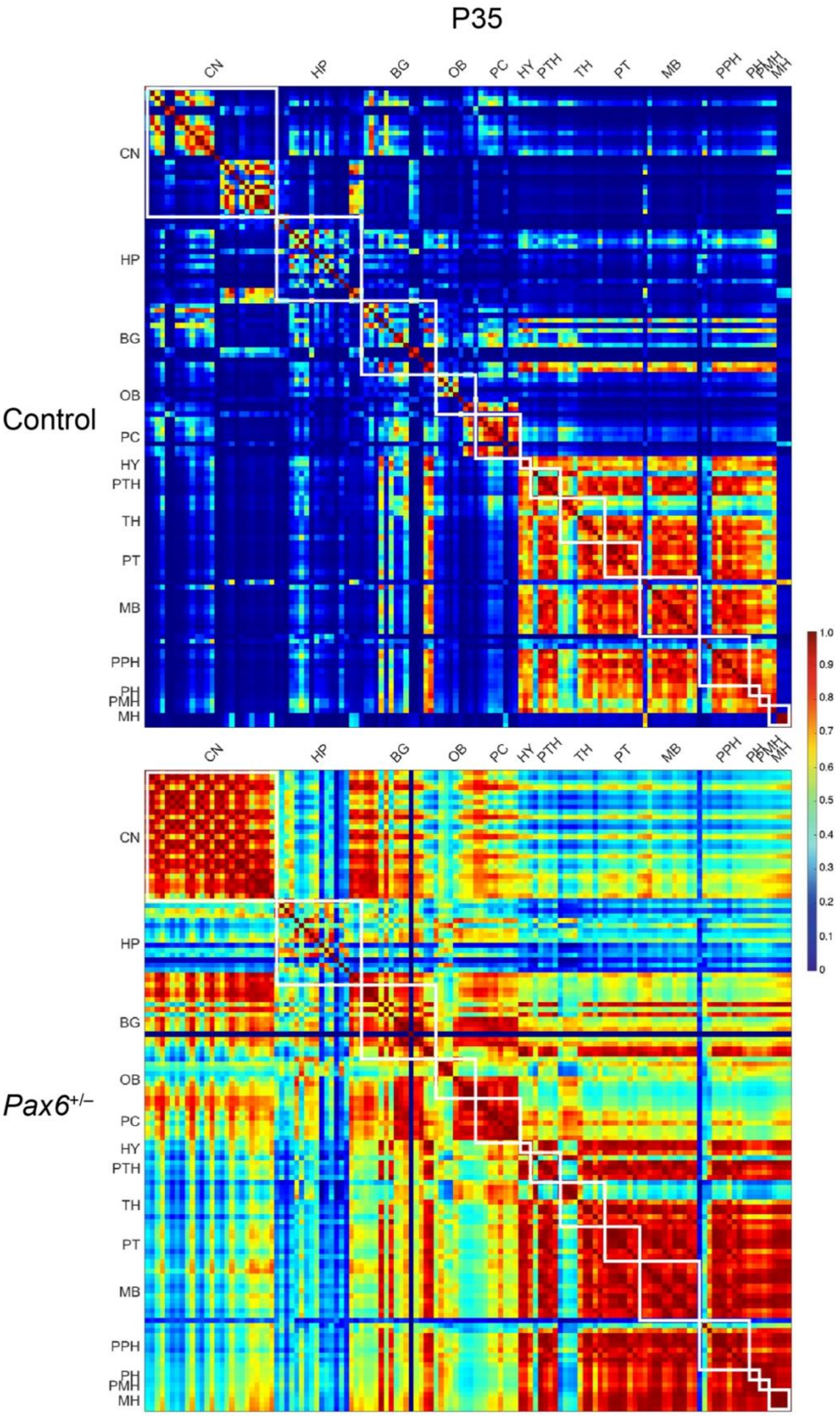
P35
Control
Pax6+/–
CN
HP
BG
OB
PC
HY
PTH
TH
PT
MB
PPH
PH
PMH
MH
1.0
0.9
0.8
0.7
0.6
0.5
0.4
0.3
0.2
0.1
0

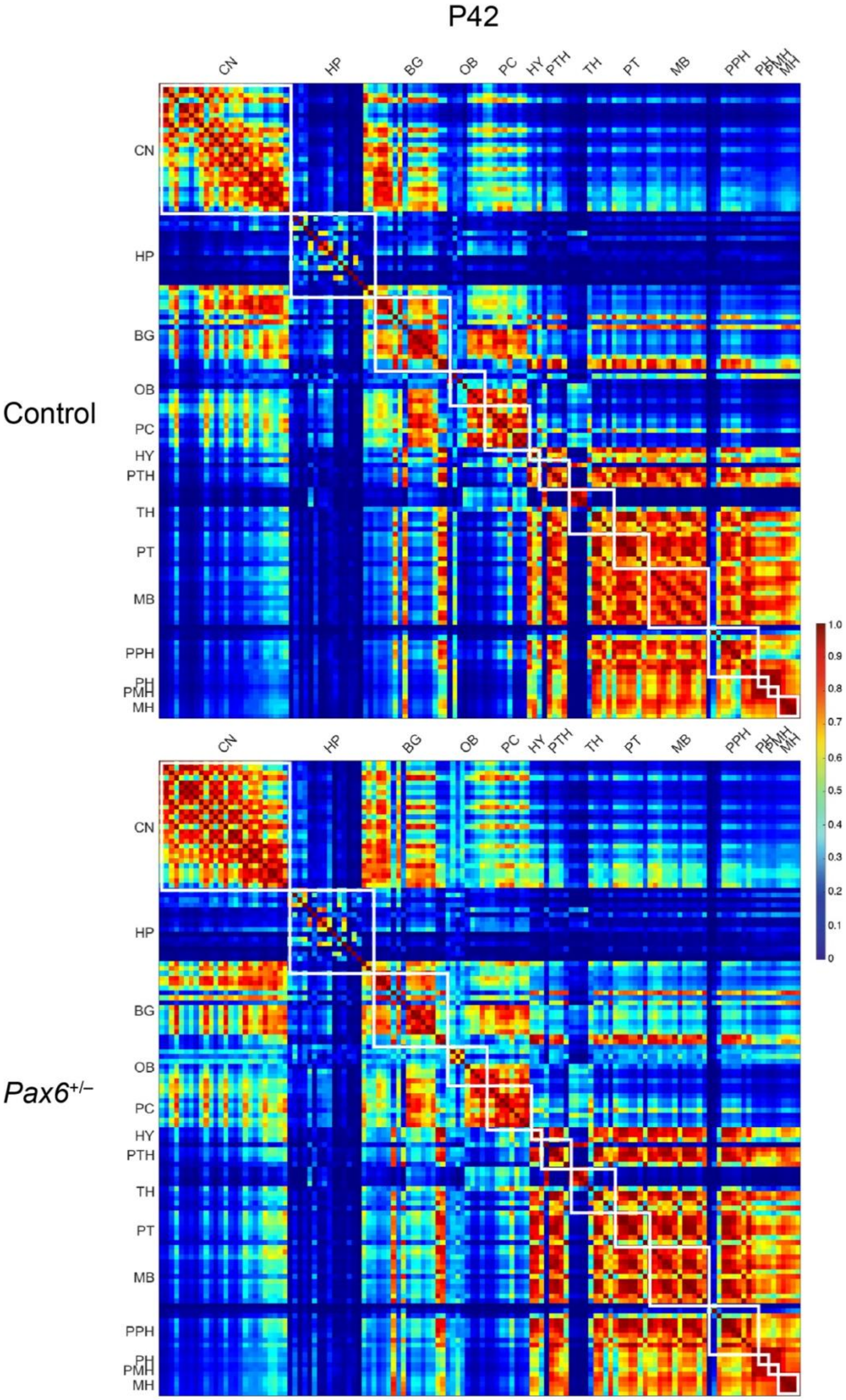

P42
Control
Pax6+/−
CN
HP
BG
OB
PC
HY
PTH
TH
PT
MB
PPH
PH
PMH
MH
1.0
0.9
0.8
0.7
0.6
0.5
0.4
0.3
0.2
0.1
0

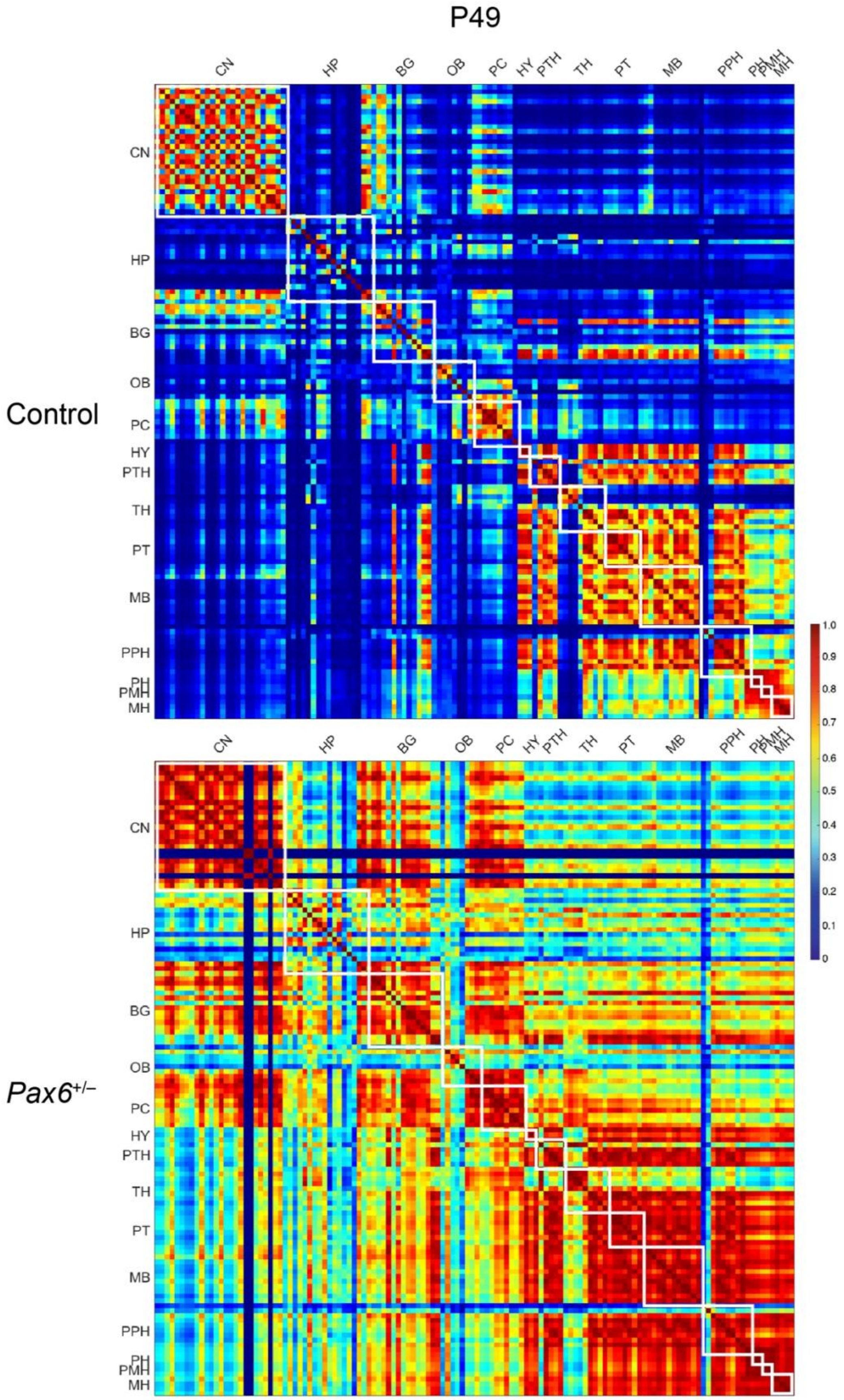

P49
Control
Pax6+/–
CN
HP
BG
OB
PC
HY
PTH
TH
PT
MB
PPH
PH
PMH
MH
1.0
0.9
0.8
0.7
0.6
0.5
0.4
0.3
0.2
0.1
0

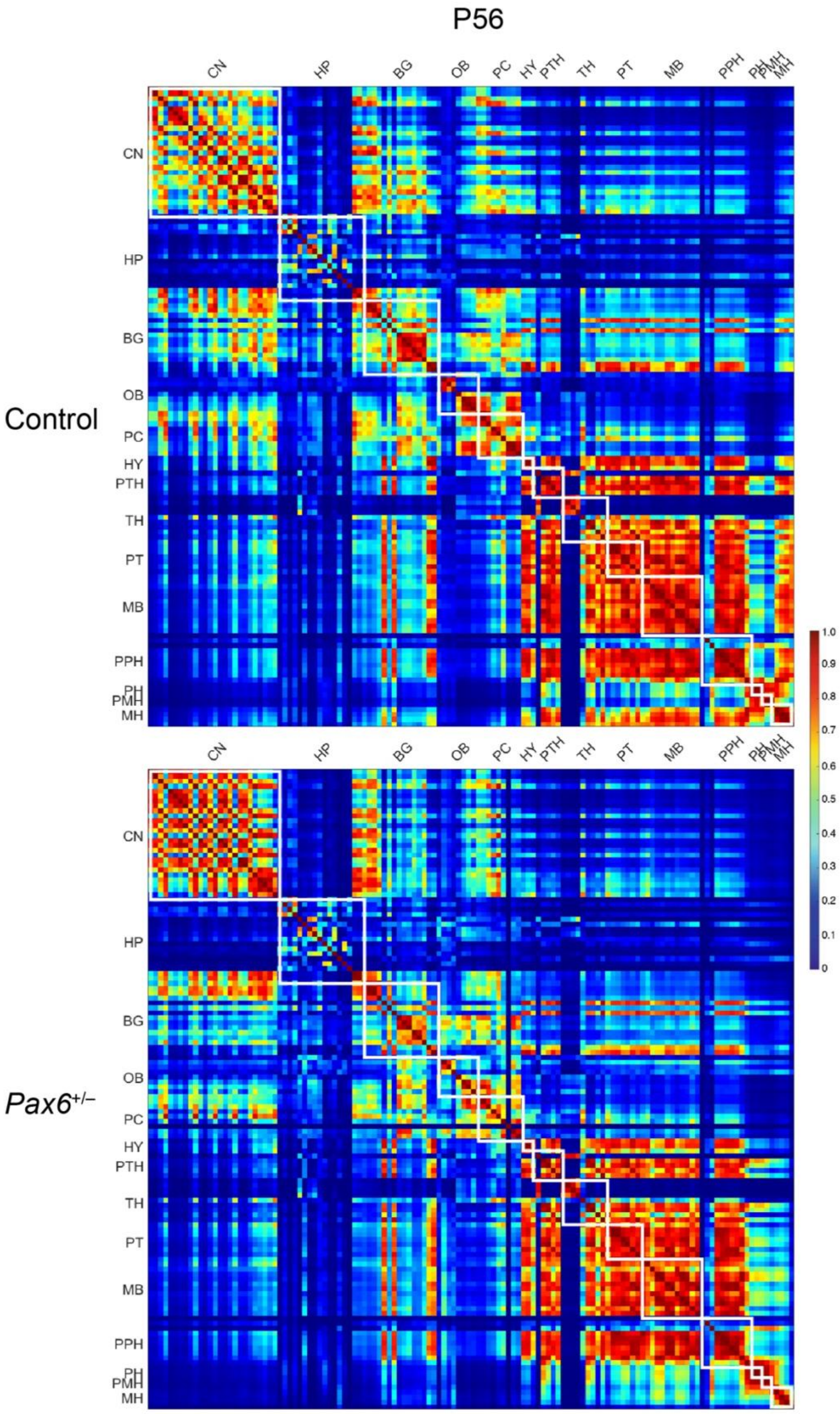
P56
Control
Pax6+/−
CN
HP
BG
OB
PC
HY
PTH
TH
PT
MB
PPH
PH
PMH
MH
1.0
0.9
0.8
0.7
0.6
0.5
0.4
0.3
0.2
0.1
0

Figure S2. Subregion similarity matrices reveal transient impairment and recovery of network properties in the *Pax6*$^{+/-}$ brain.

Matrix heatmaps of similarities between pairs of brain subregions (rows and columns) at nine ages of control (top panels) and *Pax6*$^{+/-}$ (bottom panels) mice. White boxes indicate the subregions that belong to the same main brain region (see region list in Supplementary Data 1). Scale bar, similarity values.

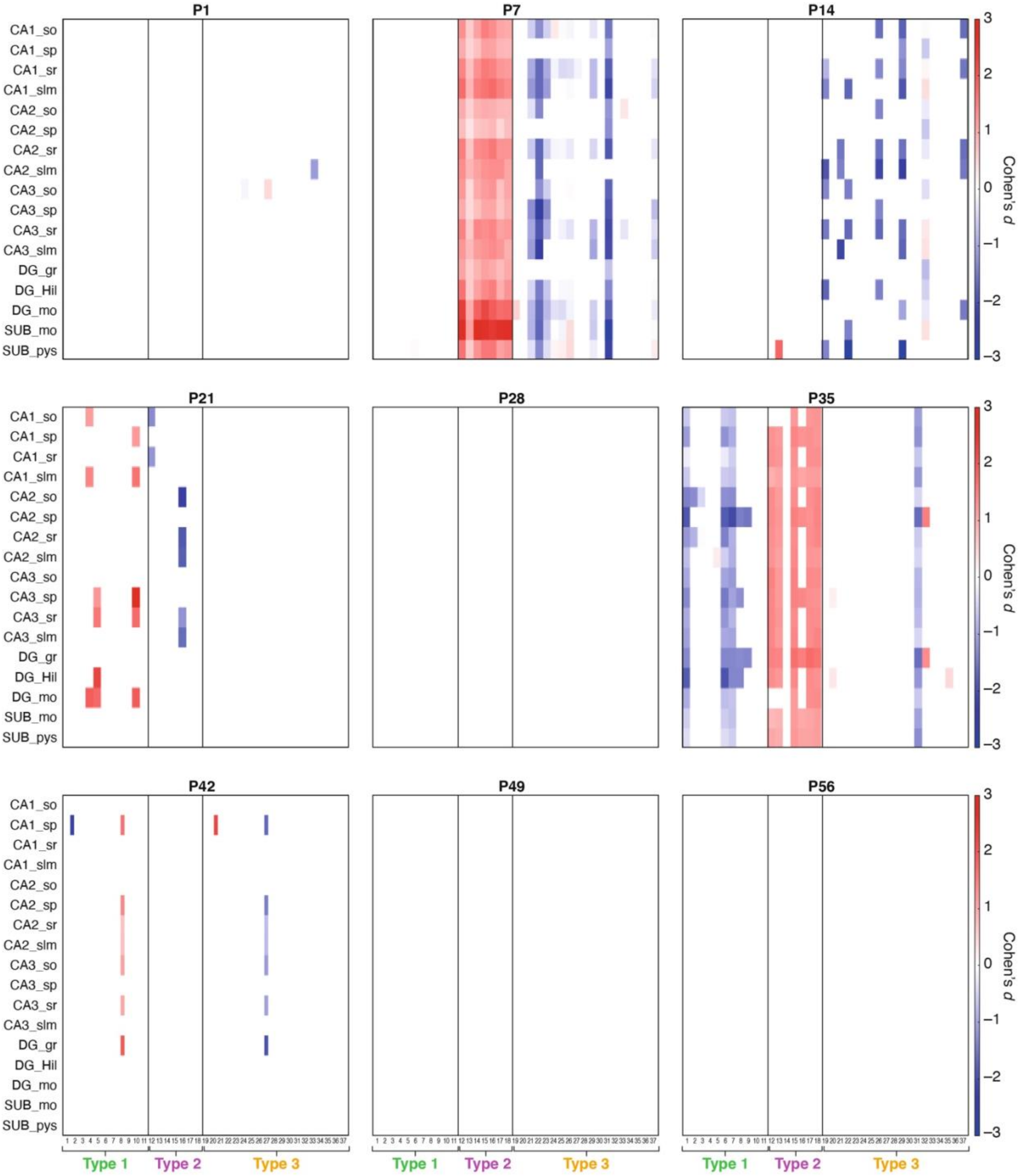

Figure S3. Hippocampal synaptome phenotypes transiently emerge and repair during postnatal development of *Pax6*$^{+/-}$ mice.

The difference (Cohen's *d*) in synapse type and subtype density in 17 hippocampal subregions at nine ages between birth and maturity between *Pax6*$^{+/-}$ and control mice. Significantly different (P<0.05, Bayesian test, Benjamini-Hochberg corrected) subregions are shown.

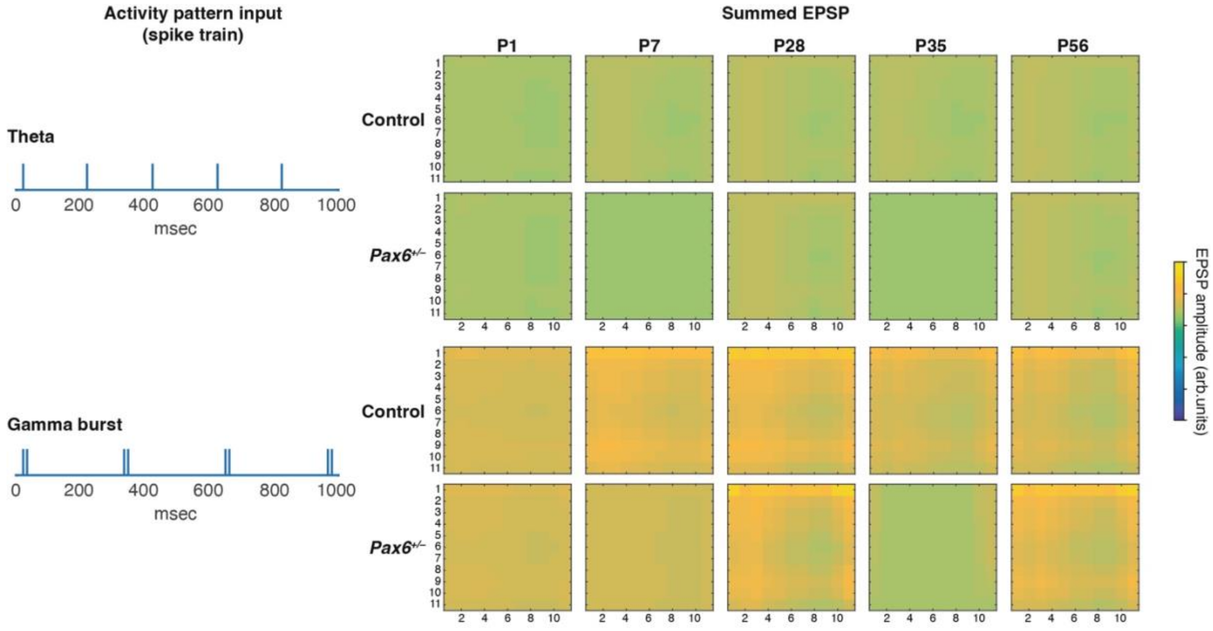

Figure S4. The impact of *Pax6* mutation on CA1 synaptic response is restricted to particular patterns of neuronal activity.

Synaptic amplitudes of age groups (P1-P56) and genotype (control, *Pax6*$^{+/-}$) were scaled based on intensity (Supplementary Data 14, 15). Synapses were activated by physiological spike patterns representing theta (top) and gamma burst (bottom) activity. Summed EPSP response amplitudes were quantified (color bar, arbitrary units). No statistically significant differences were observed between the synaptic responses of control (upper) and *Pax6*$^{+/-}$ (lower) mice to these two spike patterns (two-sided t-test, Benjamini-Hochberg corrected, N=121 samples).
