# Supplementary material for: Developmental disruption and restoration of brain synaptome architecture in the murine Pax6 neurodevelopmental disease model
Source: Nat Commun. 2022 Nov 11;13:6836. doi: 10.1038/s41467-022-34131-w (PMC9652404; doi:10.1038/s41467-022-34131-w)
Supplement: Supplementary file 3 — Description of Additional Supplementary Files [file 41467_2022_34131_MOESM3_ESM.pdf]

Description of Additional Supplementary Files

Supplementary Data 1.
Description: Names of brain regions and subregions in heatmaps.
The identity of 131 subregions and their order used in heatmaps and similarity matrices. https://doi.org/10.7488/ds/3770

File Name: Supplementary Data 2.
Description: PSD95 synaptome parameters in different brain subregions.
Column A represents the 131 brain subregions analyzed and the first row the mouse ID number. PSD95 punctum density, intensity and size values in different brain subregions as defined in the Developing Allen Reference Atlas. Each spreadsheet contains data for each PSD95 parameter at one time point (P1, P7, P14, P21, P28, P35, P42, P49, P56). Abbreviations, see Supplementary Data 1.

File Name: Supplementary Data 3.
Description: SAP102 synaptome parameters in different brain subregions.
Column A represents the 131 brain subregions analyzed and the first row the mouse ID number. SAP102 punctum density, intensity and size values in different brain subregions as defined in the Developing Allen Reference Atlas. Each spreadsheet contains data for each SAP102 parameter at one time point (P1, P7, P14, P21, P28, P35, P42, P49, P56). Abbreviations, see Supplementary Data 1.

File Name: Supplementary Data 4.
Description: Colocalization of synaptome parameters in different brain subregions.
Column A represents the 131 brain subregions analyzed and the first row the mouse ID number. Colocalization values in different brain subregions as defined in the Developing Allen Reference Atlas. Each spreadsheet contains colocalization data at one time point (P1, P7, P14, P21, P28, P35, P42, P49, P56). Abbreviations, see Supplementary Data 1.

File Name: Supplementary Data 5.
Description: Subtype classification in different brain subregions at P1.

Column A represents the 131 brain subregions, the first row corresponds to the mouse ID number and the third row the number of the subtype. Subtype classification values in different brain subregions as defined in the Developing Allen Reference Atlas at P1. Each spreadsheet contains subtype classification data from each subtype (1-37). Abbreviations, see Supplementary Data 1.

File Name: Supplementary Data 6.
Description: Subtype classification in different brain subregions at P7.
Column A represents the 131 brain subregions, the first row corresponds to the mouse ID number and the third row the number of the subtype. Subtype classification values in different brain subregions as defined in the Developing Allen Reference Atlas at P7. Each spreadsheet contains subtype classification data from each subtype (1-37). Abbreviations, see Supplementary Data 1.

File Name: Supplementary Data 7.
Description: Subtype classification in different brain subregions at P14.
Column A represents the 131 brain subregions, the first row corresponds to the mouse ID number and the third row the number of the subtype. Subtype classification values in different brain subregions as defined in the Developing Allen Reference Atlas at P14. Each spreadsheet contains subtype classification data from each subtype (1-37). Abbreviations, see Supplementary Data 1.

File Name: Supplementary Data 8.
Description: Subtype classification in different brain subregions at P21.
Column A represents the 131 brain subregions, the first row corresponds to the mouse ID number and the third row the number of the subtype. Subtype classification values in different brain subregions as defined in the Developing Allen Reference Atlas at P21. Each spreadsheet contains subtype classification data from each subtype (1-37). Abbreviations, see Supplementary Data 1.

File Name: Supplementary Data 9.
Description: Subtype classification in different brain subregions at P28.
Column A represents the 131 brain subregions, the first row corresponds to the mouse ID number and the third row the number of the subtype. Subtype classification values

in different brain subregions as defined in the Developing Allen Reference Atlas at P28. Each spreadsheet contains subtype classification data from each subtype (1-37). Abbreviations, see Supplementary Data 1.

File Name: Supplementary Data 10.

Description: Subtype classification in different brain subregions at P35.

Column A represents the 131 brain subregions, the first row corresponds to the mouse ID number and the third row the number of the subtype. Subtype classification values in different brain subregions as defined in the Developing Allen Reference Atlas at P35. Each spreadsheet contains subtype classification data from each subtype (1-37). Abbreviations, see Supplementary Data 1.

File Name: Supplementary Data 11.

Description: Subtype classification in different brain subregions at P42.

Column A represents the 131 brain subregions, the first row corresponds to the mouse ID number and the third row the number of the subtype. Subtype classification values in different brain subregions as defined in the Developing Allen Reference Atlas at P42. Each spreadsheet contains subtype classification data from each subtype (1-37). Abbreviations, see Supplementary Data 1.

File Name: Supplementary Data 12.

Description: Subtype classification in different brain subregions at P49.

Column A represents the 131 brain subregions, the first row corresponds to the mouse ID number and the third row the number of the subtype. Subtype classification values in different brain subregions as defined in the Developing Allen Reference Atlas at P49. Each spreadsheet contains subtype classification data from each subtype (1-37). Abbreviations, see Supplementary Data 1.

File Name: Supplementary Data 13.

Description: Subtype classification in different brain subregions at P56.

Column A represents the 131 brain subregions, the first row corresponds to the mouse ID number and the third row the number of the subtype. Subtype classification values in different brain subregions as defined in the Developing Allen Reference Atlas at

P56. Each spreadsheet contains subtype classification data from each subtype (1-37). Abbreviations, see Supplementary Data 1.

File Name: Supplementary Data 14.
Description: PSD95 gradients in the CA1sr of the hippocampus.
The PSD95 density, intensity and size parameters along the radial and tangential axis in the CA1 striatum radiatum subregion of the hippocampus. Each column contains the PSD95 values of individual mice. Each spreadsheet contains data for each PSD95 parameter at one time point (P1, P7, P14, P21, P28, P35, P42, P49, P56).

File Name: Supplementary Data 15.
Description: SAP102 gradients in the CA1sr of the hippocampus.
The SAP102 density, intensity and size parameters along the radial and tangential axis in the CA1 striatum radiatum subregion of the hippocampus. Each column contains the SAP102 values of individual mice. Each spreadsheet contains data for each PSD95 parameter at one time point (P1, P7, P14, P21, P28, P35, P42, P49, P56).

File Name: Supplementary Data 16.
Description: Synapse subtype phenotypes in the neocortex of *Pax6*$^{+/-}$ mice.
Column A, time points (P7, P14, P21, P35, P42) with significant differences; column B, neocortical areas as defined in the Developing Allen Reference Atlas at P56. Abbreviations, see Supplementary Data 1; columns C-AM, 37 synapse subtypes with differences (Q-values) between control and *Pax6*$^{+/-}$ mice shown. Red shading indicates significance: dark red Q<0.001, medium red Q<0.01, pale red Q<0.05.
