# Supplementary material for: Developmental disruption and restoration of brain synaptome architecture in the murine Pax6 neurodevelopmental disease model
Source: Nat Commun. 2022 Nov 11;13:6836. doi: 10.1038/s41467-022-34131-w (PMC9652404; doi:10.1038/s41467-022-34131-w)
Supplement: Supplementary file 20 — Reporting Summary [file 41467_2022_34131_MOESM20_ESM.pdf]

nature portfolio

Corresponding author(s): Seth G.N. Grant

Last updated by author(s): Oct 5, 2022

Reporting Summary

Nature Portfolio wishes to improve the reproducibility of the work that we publish. This form provides structure for consistency and transparency in reporting. For further information on Nature Portfolio policies, see our Editorial Policies and the Editorial Policy Checklist.

Statistics

For all statistical analyses, confirm that the following items are present in the figure legend, table legend, main text, or Methods section.

n/a	Confirmed	
☐	☒	The exact sample size (*n*) for each experimental group/condition, given as a discrete number and unit of measurement
☐	☒	A statement on whether measurements were taken from distinct samples or whether the same sample was measured repeatedly
☐	☒	The statistical test(s) used AND whether they are one- or two-sided<br>*Only common tests should be described solely by name; describe more complex techniques in the Methods section.*
☐	☒	A description of all covariates tested
☐	☒	A description of any assumptions or corrections, such as tests of normality and adjustment for multiple comparisons
☒	☐	A full description of the statistical parameters including central tendency (e.g. means) or other basic estimates (e.g. regression coefficient) AND variation (e.g. standard deviation) or associated estimates of uncertainty (e.g. confidence intervals)
☐	☒	For null hypothesis testing, the test statistic (e.g. *F*, *t*, *r*) with confidence intervals, effect sizes, degrees of freedom and *P* value noted<br>*Give P values as exact values whenever suitable.*
☐	☒	For Bayesian analysis, information on the choice of priors and Markov chain Monte Carlo settings
☒	☐	For hierarchical and complex designs, identification of the appropriate level for tests and full reporting of outcomes
☐	☒	Estimates of effect sizes (e.g. Cohen's *d*, Pearson's *r*), indicating how they were calculated

Our web collection on statistics for biologists contains articles on many of the points above.

Software and code

Policy information about availability of computer code

Data collection	All software used in this study is described and published in Cizeron et al, Science 369, 270-275. doi: 10.1126/science.aba3163 and Zhu et al Neuron 99, 781-799. https://doi.org/10.1016/j.neuron.2018.07.007
Data analysis	Matlab 2014b, Matlab2019a, Pycharm Community version 2018.1.3 Origin 2019b. All software used in this study is described and published in Cizeron et al, Science 369, 270-275. doi: 10.1126/science.aba3163 and Zhu et al Neuron 99, 781-799. https://doi.org/10.1016/j.neuron.2018.07.007

For manuscripts utilizing custom algorithms or software that are central to the research but not yet described in published literature, software must be made available to editors and reviewers. We strongly encourage code deposition in a community repository (e.g. GitHub). See the Nature Portfolio guidelines for submitting code & software for further information.

Data

Policy information about availability of data

All manuscripts must include a data availability statement. This statement should provide the following information, where applicable:

- Accession codes, unique identifiers, or web links for publicly available datasets
- A description of any restrictions on data availability
- For clinical datasets or third party data, please ensure that the statement adheres to our policy

The synaptic data generated in this study have been deposited at Edinburgh DataShare (https://doi.org/10.7488/ds/3770) and project website (https://brain-synaptome.org/Pax6_developmental_synaptome_atlas). There are no restrictions on data availability.

Field-specific reporting

Please select the one below that is the best fit for your research. If you are not sure, read the appropriate sections before making your selection.

☒ Life sciences ☐ Behavioural & social sciences ☐ Ecological, evolutionary & environmental sciences

For a reference copy of the document with all sections, see nature.com/documents/nr-reporting-summary-flat.pdf

Life sciences study design

All studies must disclose on these points even when the disclosure is negative.

Sample size	We estimated the sample size according to our previous studies in mutant and wild type mice(Cizeron et al., 2020; Zhu et al., 2018) using a T-test analysis. The sample size for each time point is specified in the Material and Methods, section Animals.
Data exclusions	We only excluded animals with absent or damaged brain regions due to errors in dissection. After imaging we only excluded images that were out of focus. We did not exclude animals after computational replication.
Replication	We performed a single large-scale experiment using cohorts of mice at 9 ages group for two genotypes. Control mice in this study showed similar results to published reports (Zhu et al, 2018; Cizeron et al, 2020) using the same methodology. Sample size was sufficient to confirm reproducibility. 142 mouse brains were collected, processed and sectioned following the same protocols and conditions detailed in the Methods sections: Tissue Collection and Sectioning; Tissue Preparation. One parasagittal section of each mouse, corresponding to the same brain anatomical level, was imaged with the same imaging protocol and parameter acquisition settings as described in the Methods, section Spinning Disk Confocal Microscopy.
Randomization	Samples were allocated according to their postnatal day of collection into 9 groups (P1-P56). When collected each sample received a randomized ID number, masking their genotypes.
Blinding	The masking ID number was maintained during tissue and imaging processing.

Reporting for specific materials, systems and methods

We require information from authors about some types of materials, experimental systems and methods used in many studies. Here, indicate whether each material, system or method listed is relevant to your study. If you are not sure if a list item applies to your research, read the appropriate section before selecting a response.

Materials & experimental systems

n/a	Involved in the study	
☒	☐	Antibodies
☒	☐	Eukaryotic cell lines
☒	☐	Palaeontology and archaeology
☐	☒	Animals and other organisms
☒	☐	Human research participants
☒	☐	Clinical data
☒	☐	Dual use research of concern

Methods

n/a	Involved in the study	
☒	☐	ChIP-seq
☒	☐	Flow cytometry
☒	☐	MRI-based neuroimaging

Animals and other organisms

Policy information about studies involving animals; ARRIVE guidelines recommended for reporting animal research

Laboratory animals	Both control (c) and mutant (m) mice were on a C57Bl6/J bacground from both sex were collected at nine postnatal time points:one (P1, c=11, m=6), seven (P7, c=7, m=7), fourteen (P14, c=6, m=7), twenty-one (P21, c=7, m=8), twenty-eight (P28, c=8, m=7), thirty-five (P35, c=11, m=6), forty-two (P42, c=7, m=6), forty-nine (P49, c=6, m=6) and fifty-six (P56, c=16, m=9) days.
Wild animals	The study did not involve wild animals.
Field-collected samples	The study did not involve samples collected from the field.
Ethics oversight	Animal procedures were performed in accordance with the Edinburgh University Animal Welfare and Ethical Review Body (AWERB PL16-19) and UK Home Office regulations.

Note that full information on the approval of the study protocol must also be provided in the manuscript.
